# High Precision Positioning and Rotation Angle Estimation of a Flatbed Truck Based on BDS and Vision

**DOI:** 10.3390/s24061826

**Published:** 2024-03-12

**Authors:** Xinli Yu, Yufei Ren, Xiaoxv Yin, Deqiang Meng, Haikuan Zhang

**Affiliations:** Qilu Aerospace Information Research Institute, Jinan 250100, China; yuxinli@aircas.ac.cn (X.Y.); yinxiaoxu2017@163.com (X.Y.); mengdq@aircas.ac.cn (D.M.); zhanghk@aircas.ac.cn (H.Z.)

**Keywords:** unmanned forklift, automated loading, feature construction, BDS and vision localization, rotation angle estimation

## Abstract

Centimeter-level localization and precise rotation angle estimation for flatbed trucks pose significant challenges in unmanned forklift automated loading scenarios. To address this issue, the study proposed a method for high-precision positioning and rotation angle estimation of flatbed trucks using the BeiDou Navigation Satellite System (BDS) and vision technology. First, an unmanned forklift equipped with a Time-of-Flight (ToF) camera and a dual-antenna mobile receiver for BDS positioning collected depth images and localization data near the front and rear endpoints of the flatbed. The Deep Dual-Resolution Network-23-slim (DDRNet-23-slim) model was used to segment the flatbed from the depth image and extract the straight lines at the edges of the flatbed using the Hough transform. The algorithm then computed the set of intersection points of the lines. A neighborhood feature vector was designed to identify the endpoint of a flatbed from a set of intersection points using feature screening. Finally, the relative coordinates of the endpoints were converted to a customized forklift navigation coordinate system by BDS positioning. A rotation angle estimation was then performed using the endpoints at the front and rear. Experiments showed that the endpoint positioning error was less than 3 cm, and the rotation angle estimation error was less than 0.3°, which verified the validity and reliability of the method.

## 1. Introduction

With the rapid development of e-commerce and supply chain management, the logistics industry is facing the requirement of being more efficient and intelligent. In warehouse logistics, automated truck loading is a key part of improving efficiency, reducing costs, and avoiding the risks associated with manual operations [1,2,3]. The accurate positioning and rotation angle estimation of flatbed trucks pose a challenge due to the uncertainty associated with manually parking trucks [4] and the complexity of various types of flatbed trucks.

Several researchers have already studied the positioning of trucks in the automatic loading process. Positioning sensors can be categorized into two types based on their installation position: fixed sensors, which are installed in specific positions [4,5,6,7], and semi-free sensors, which are mounted on stationary devices such as conveyor belts [8,9,10]. Positioning based on a single fixed sensor results in large errors due to the long observation distances. Positioning based on multiple fixed sensors or semi-free sensors can meet the demand for pallet loading, but it requires drivers to have better parking skills. Conveyor belts and other fixed installations have a high level of automation and standardization. However, this scheme is more suitable for newly built logistics parks due to the high cost associated with retrofitting old logistics parks that did not consider automation and intelligence during the initial construction phase.

Free sensors, those mounted on unmanned forklifts, are often used for localizing the forklift itself [11], as well as for identifying and locating pallets [12,13] during the loading and unloading of trucks. Limited research has been conducted on the localization of trucks using visual sensors mounted on forklifts. However, this positioning method offers the advantages of cost-effectiveness, high accuracy, and low requirements for truck driver parking skills.

This study aims to explore and address the issue of flatbed truck localization and rotation angle estimation in the automated loading process. Combining the advantages and disadvantages of the aforementioned sensors, we have developed a high-precision and stable method for positioning and estimating the corners of flatbed trucks. The advantage of this method is that it does not require human intervention or installation of fixtures, making it conducive to achieving intelligent loading in older logistics parks. It is compatible with outdoor all-day operation scenarios and allows for the automatic loading of flatbed trucks of varying lengths.

The main innovations are as follows: (1) Visual sensors and BeiDou Navigation Satellite System (BDS) dual-antenna receivers were installed on unmanned forklifts to enable flatbed truck positioning. The shorter measuring distance and high-precision BDS positioning have enhanced the accuracy of flatbed truck positioning. (2) Endpoint neighborhood feature vectors were designed, and endpoint pixel coordinates were accurately obtained using the feature screening method. This approach is beneficial for enhancing the positioning accuracy of flatbed trucks.

In this paper, a review of truck localization and measurement methods related to automated truck loading is presented. The detailed steps for designing and implementing our proposed method are presented in Section 2. Furthermore, experiments are conducted to validate the practicality and applicability of this method in Section 3 and Section 4. Finally, the study concludes by discussing its findings, limitations, and future directions for expansion in Section 5.

## 2. Method

**Figure 1:**
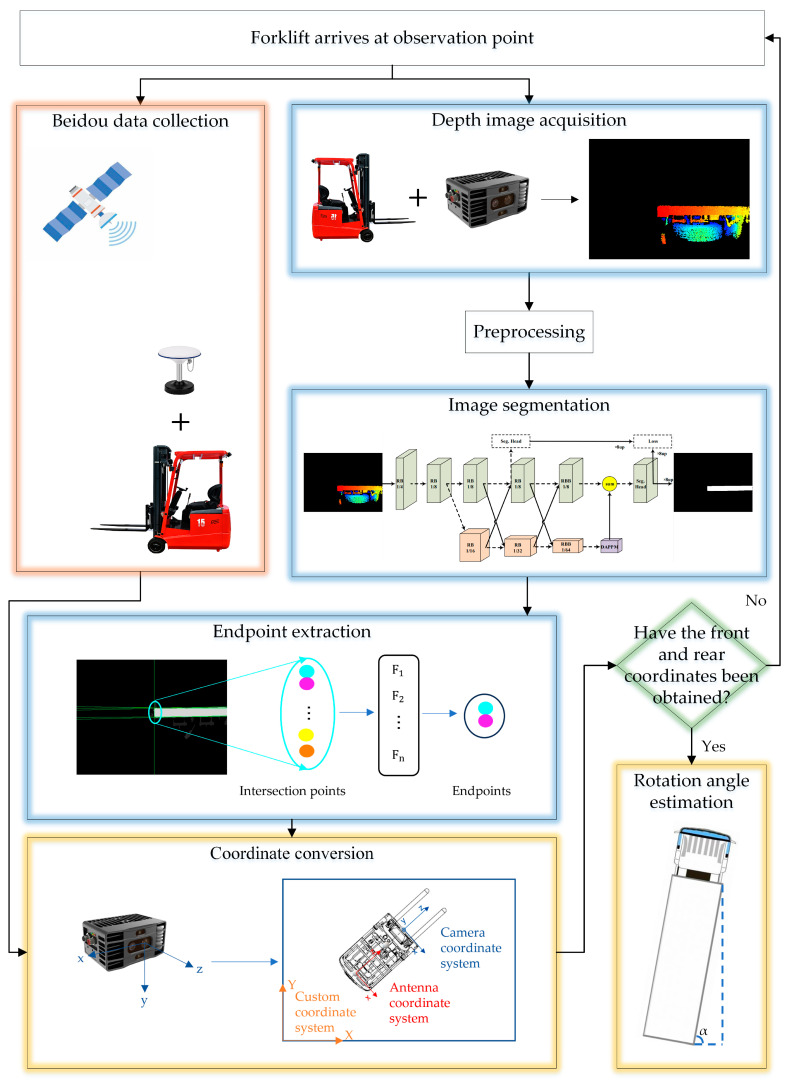
Flowchart of the overall methodology.

Our method can be divided into five modules: depth image acquisition and preprocessing, image segmentation, endpoint extraction, BDS data acquisition, and coordinate conversion with rotation angle estimation. Firstly, the double-antenna mobile receiver was installed on the unmanned forklift to acquire the position and yaw angle of the forklift. Additionally, an RGB-D camera was installed to capture depth images near the front and rear endpoints of the flatbed. The truck flatbed was segmented from the depth image using the Deep Dual-Resolution Network-23-slim (DDRNet-23-slim) [14]. Straight lines were extracted from the edges of the flatbed using the Hough transform [15], and the intersection sets of these lines were computed. A neighborhood feature vector was subsequently designed to locate the endpoint of a flatbed from a set of intersection points through feature screening. Finally, the relative coordinates of endpoints were converted to the absolute coordinate system using the BDS positioning. This process determined the precise position of the flatbed truck, and the rotation angle estimation was performed based on the front and rear endpoints. The flowchart is shown in Figure 1.

### 2.1. Depth Image Acquisition and Preprocessing

An unmanned forklift equipped with a Time-of-Flight (ToF) camera receives a loading task, travels to the first observation point to observe, records the results, and then moves to the second observation point for further observation. The unmanned forklift’s movement path from the first observation point to the second observation point was planned by an autonomous path planning algorithm integrated into the forklift. The navigation mode was BDS fusion inertial combination navigation.

**Figure 2:**
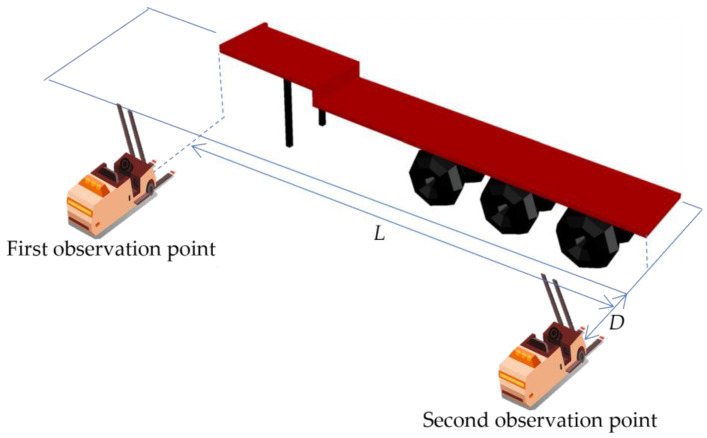
Schematic diagram of observation points.

The location of the observation point is related to the length of the flatbed, the parking space line, the camera’s view and ranging accuracy, the length of the fork, and the forklift motion control error. Flatbed trucks were backing into parking spaces, and their rearward movement was limited by the placement of wheel chocks. The second observation point was located along the extension of the rear parking space line. The distance from the first observation point to the second observation point along the side parking space line is equal to the length of the flatbed. The vertical distance between the observation points and the near sideline of the parking primarily depends on the camera’s viewing angle and ranging accuracy. It must meet the following conditions: (1) One endpoint of the flatbed must be within the camera’s view, even if the vehicle is parked at an angle, and there are errors in the forklift motion control. (2) Observation points should not be located too close to flatbed trucks to prevent safety accidents. (3) To maximize ranging accuracy, the observation point should not be too far away from the truck’s position line because ranging accuracy decreases with distance. The location of the observation point is determined artificially by combining the above conditions. The details of the observation point, loading truck’s space number, type, flatbed length, and loading task are provided to the forklift. Among them, the second observation point is associated with the parking space number, while the first observation point is determined by the second observation point and the length of the flatbed. The distribution of observation points is shown in Figure 2, where L denotes the length of the flatbed and D denotes the vertical distance between the observation points and the near sideline of the parking.

To eliminate interfering pixels in the depth image, the segmentation threshold T was set to the maximum depth of the target. By removing depth data larger than T, the segmented image should contain the target, with most of the non-target depth data eliminated. The threshold T is greater than the distance D from the observation point to the near parking sideline but less than the distance from the observation point to the other parking sideline.

The manufacturing accuracy and assembly deviation of the lens lead to the distortion of the original image [16], which needs to be corrected using the internal and external parameters of the camera. The depth jitter at the edges of the object is strong and there is noise interference on the depth map. Therefore, Gaussian filtering on a large scale was applied to smooth the image and reduce the noise.

### 2.2. Image Segmentation

**Figure 3:**
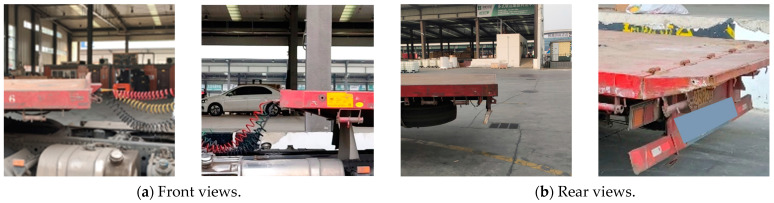
Front and rear views of flatbed trucks with different styles.

For a wide variety of flatbed trucks (Figure 3), coupled with the inconsistent degree of deformation due to use, traditional methods of image processing are difficult to be compatible with these cases. Therefore, deep learning algorithms were used for image segmentation.

Classical semantic segmentation networks typically prioritize high accuracy at the expense of heavy computation and long inference times. However, for unmanned forklifts, minimizing inference time is crucial, and excessive computation is undesirable. DDRNet-23-slim introduces deep high-resolution real-time semantic segmentation, achieving a balance between speed and accuracy by increasing the model’s width and depth [14]. The architecture of the DDRNet-23-slim is shown in Table 1.

**Figure 4:**
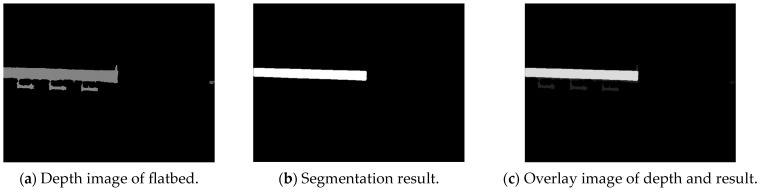
Segmentation results.

The segmentation results are shown in Figure 4.

After segmenting out the flatbed, the depth image was masked to retain only the depth information of the flatbed region. Afterwards, the non-null values in the masked depth image were set to 255, while the null values were set to 0 to generate the binary depth image.

### 2.3. Endpoint Extraction

Edge detection is a common method for segmenting images based on gray-level variations, which essentially involves extracting the features of the discontinuous parts of the image [17]. Canny edge extraction [17] was performed on the depth binary image, followed by the extraction of straight lines using the Hough transform [15]. The lines that were identified as approximately horizontal and vertical, i.e., lines with angles close to 0 and 90 degrees to the upward direction, were labeled as set $A_{T}$ and set $B_{T}$, respectively. The intersection of the lines in set $A_{T}$ with the lines in set $B_{T}$ was then calculated. The set of intersection pixels was denoted as J.

**Figure 5:**
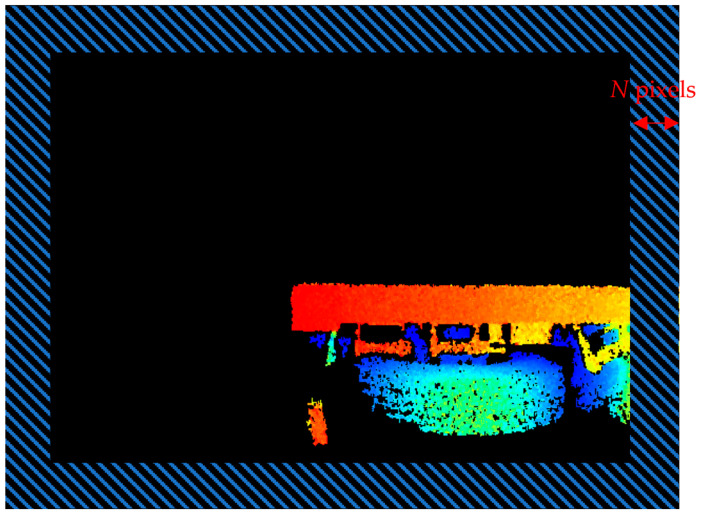
Schematic of the image edge area.

The intersection point should be located at a specific distance of N pixels from the edge. Otherwise, the subsequent intersection screening process is not possible. Therefore, the intersection points that fall within the shaded area shown in Figure 5 were eliminated. The remaining set of intersection pixels was denoted as $J_{1}$. The depth of the flatbed truck in Figure 5 was illustrated using color bars, with red representing the closest end and blue indicating the farthest end.

**Figure 6:**
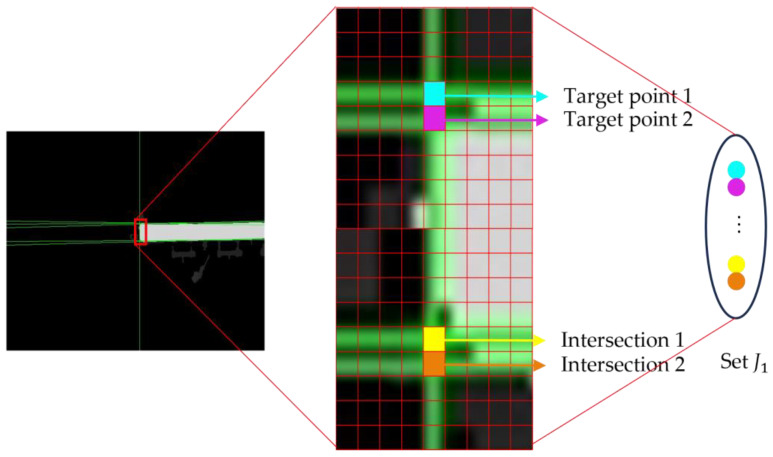
Schematic diagram of the intersection set.

The set $J_{1}$ contains endpoints and some non-endpoints as shown in Figure 6. A neighborhood template matching method was proposed for selecting the endpoints from $J_{1}$.

Depending on the observation direction, the depth image, the endpoint schematic image, and the neighborhood pixel values in the upper-left, upper-right, lower-right, and lower-left sectors of the endpoints theoretically fall into the following two cases.

**Figure 7:**
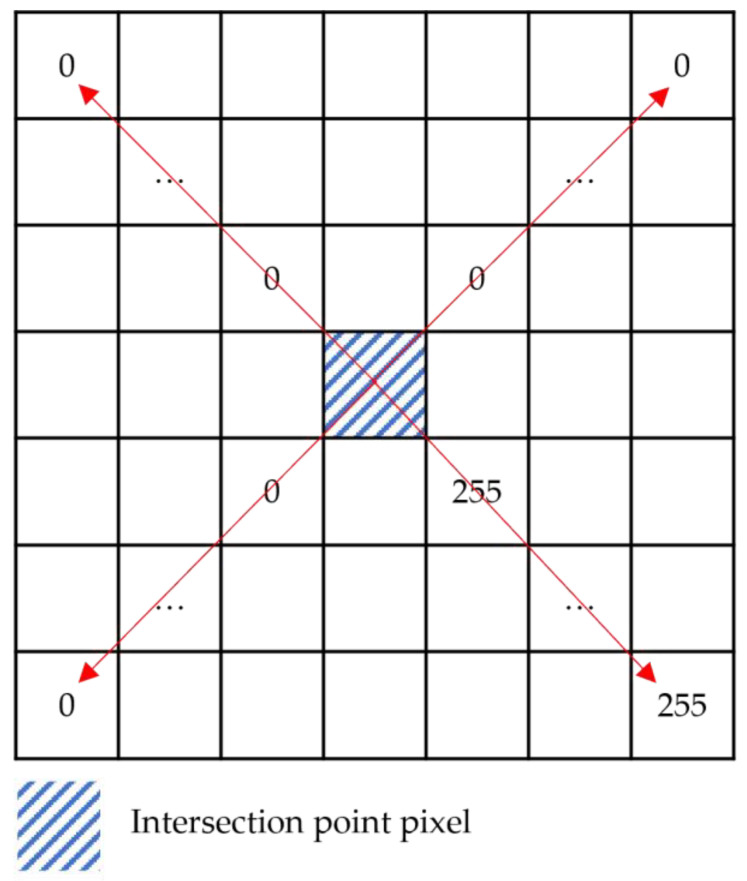
Neighborhood window.

The depth image is unstable at the boundary, resulting in endpoints that do not align precisely with the theoretical pixel features. Therefore, the statistical range of neighborhood features has been expanded. A square window with a side length of 2N+1,N∈Z pixels was constructed centered on the intersection point, as shown in Figure 7. The pixel values in the upper-left, upper-right, lower-right, and lower-left sectors of this intersection point were recorded as its features. Taking case 1 in Table 2 as an example, this intersection pixel is characterized by the vector $F=[F_{ul},F_{ur},F_{lr},F_{ll}]$, where $F_{ul}=[0,0,\ldots ,0]$, $F_{ur}=[0,0,\ldots ,0]$, $F_{lr}=[255,255,\ldots ,255]$, $F_{ll}=[0,0,\ldots ,0]$, and each vector contains N elements.

For any intersection point, if the number of elements in the $F_{ul}$, $F_{ur}$, and $F_{ll}$ vectors with a value of 255 was less than 1/5 of N and the number of elements in the $F_{lr}$ vector with a value of 255 was more than 4/5 of N, the intersection point was considered an endpoint. The set consisting of multiple endpoints was denoted as $J_{2}$. As shown in Formula (1), where $F_{ul}^{i}$, $F_{ur}^{i}$, $F_{lr}^{i}$, and $F_{ll}^{i}$ denote the ith element in the feature vectors $F_{ul}$, $F_{ur}$, $F_{ll}$, and $F_{lr}$, respectively, and N denotes the number of elements in the vector.

(1) $$\left\{\begin{array}{c} \begin{array}{c} \frac{\sum_{i=1}^{i=N}F_{ul}^{i}}{255}<N*\frac{1}{5}\\ \frac{\sum_{i=1}^{i=N}F_{ur}^{i}}{255}<N*\frac{1}{5}\\ \frac{\sum_{i=1}^{i=N}F_{lr}^{i}}{255}>N*\frac{4}{5} \end{array}\\ \frac{\sum_{i=1}^{i=N}F_{ll}^{i}}{255}<N*\frac{1}{5} \end{array}\right.$$

$J_{2}$ is an endpoint cluster where the distance between each element should be small. The average of the pixel coordinates of each element was then calculated to determine the endpoint pixel coordinates.

**Figure 8:**
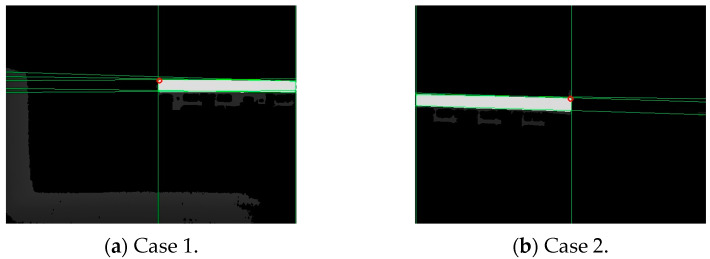
Endpoint localization results.

The depth at the endpoint obtained from the above calculation might be null, which in the binary image was represented by a value of 0. The first pixel with a non-null depth in the lower-right or lower-left direction of the neighborhood was selected as the corrected endpoint. For example, in case 1 of Table 2, the first pixel with a non-null depth in the lower-right direction of the intersection point was considered the corrected endpoint; in case 2 of Table 2, the first pixel with a non-null depth in the lower-left direction of the intersection point was considered the corrected endpoint. The corrected endpoint localization results are shown in Figure 8, where the red hollow circles indicate the corrected endpoints.

### 2.4. BDS Data Acquisition

**Figure 9:**
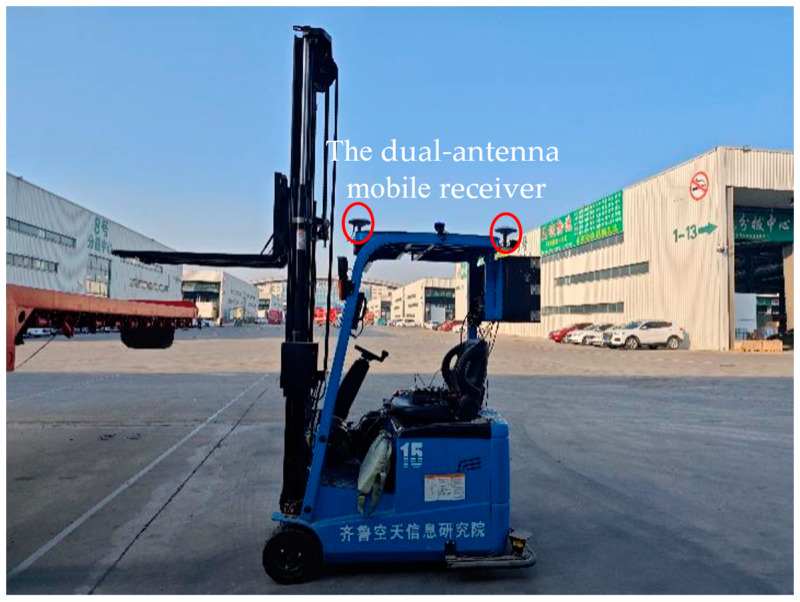
Diagram of the dual-antenna mobile receiver installation locations.

A BDS reference station was established near the logistics center, and a dual-antenna mobile receiver was mounted on top of the forklift, as illustrated in Figure 9. The data from the BDS reference station and the mobile receiver were differentially processed to eliminate errors such as atmospheric delays and receiver clock differences. This process aimed to obtain the precise position and yaw angle of the forklift.

### 2.5. Coordinate Conversion and Rotation Angle Estimation

The depth image was mapped to the point cloud data using the camera’s internal and external references. Subsequently, the spatial location of the flatbed endpoint under the camera coordinate system was determined by indexing the pixel coordinate in the depth image. To navigate effectively, the camera coordinate system must be converted to the forklift navigation coordinate system. In this paper, a custom plane coordinate system was used for forklift navigation.

The coordinates of the forklift itself in the custom coordinate system are actually the position of the main antenna, set as ($X_{h},Y_{h}$), because the forklift navigation depends on the antenna to receive signals.

Before loading the flatbed truck, the logistics warehouse management system should allocate the goods based on the flatbed truck type and load capacity. Subsequently, the type of flatbed truck and the loading task were assigned to the forklift. The height of the flatbed truck and the lifting fork during loading is determined by the type of flatbed truck. The data collected from the in-place sensor on the forklift, rather than the height of the flatbed truck, were used to determine the status of cargo stacking. Therefore, measuring the height of the flatbed truck is meaningless. Consequently, the coordinates of the target endpoint were simplified to ($x_{1},z_{1}$) by disregarding the height information.

**Figure 10:**
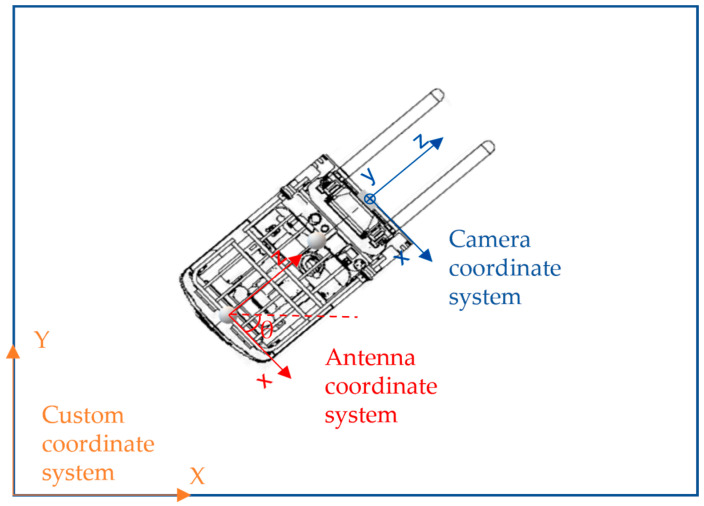
Positional relationship diagram between camera coordinate system, antenna coordinate system, and custom coordinate system.

The antenna coordinate system was established by translating the camera coordinate system after dimensionality reduction, as depicted in Figure 10. Assuming that the origin of the camera plane coordinate system is shifted to the left by △x along the X-axis direction and shifted downward by △z along the Z-axis direction to coincide with the antenna coordinate system, the coordinates of the target endpoint under the antenna coordinate system would be $(x_{1}+△x,z_{1}+△z$).

Taking the custom coordinate system mentioned above as an example, let us assume that the antenna coordinate system rotated counterclockwise by an angle θ to align with the local coordinate system. Then, the target endpoint $(x_{1}+△x,z_{1}+△z$) in the antenna coordinate system was converted to the customized coordinate system as shown in Formulas (2) and (3), where ($X_{h},Y_{h}$) represents the position of the main antenna.

(2) $$X=\left(x_{1}+△x\right)cos\theta +\left(z_{1}+△z\right)sin\theta +X_{h}$$

(3) $$Y=-\left(x_{1}+△x\right)sin\theta +\left(z_{1}+△z\right)cos\theta +Y_{h}$$

In the custom coordinate system, the front endpoint of the flatbed truck was labeled as ${(X}_{1},Y_{1})$, and the rear endpoint was labeled as ${(X}_{2},Y_{2})$. The linear Equation (4), which can be used to determine the cargo position, is constructed as follows.

(4) $$\frac{X-X_{1}}{X_{2}-X_{1}}=\frac{Y-Y_{1}}{Y_{2}-Y_{1}}$$

The truck rotation angle α was calculated as shown in Formula (5).

(5) $$\alpha =\arctan\left(\frac{Y_{1}-Y_{2}}{X_{1}-X_{2}}\right)/pi\times 180{}^{\circ}$$

**Figure 11:**
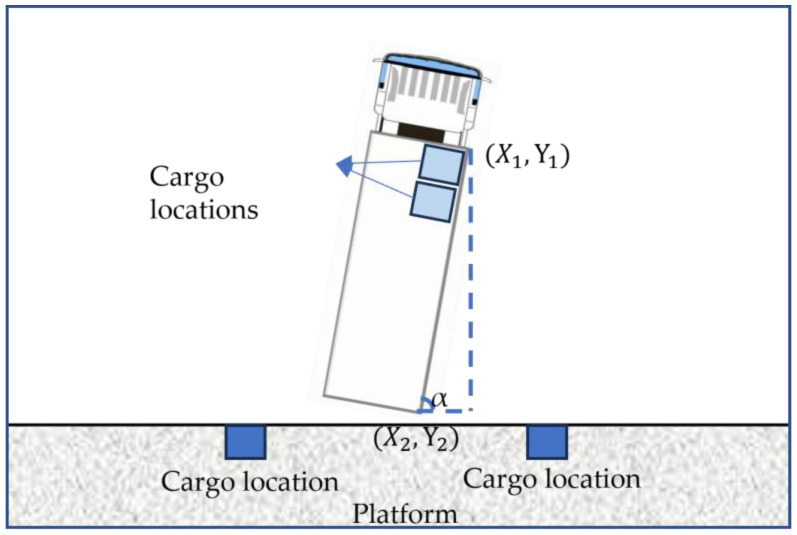
Example diagram of a rotated truck.

The flatbed truck was rotated as shown in Figure 11.

## 3. Experiments

### 3.1. Forklift and Sensor Selection

The hardware equipment used in this research includes a forklift body, a ToF camera, and a BDS dual-antenna mobile receiver. The forklift body is produced by Hefei Banyitong Technology Development Co., Ltd. in Hefei, China. The motion control precision of the unmanned forklift is 3 cm. The camera is manufactured by Shanghai Tuyang Information Technology Co., Ltd. in Shanghai, China. The camera has a range error of 9 mm at a distance of 2 m. The BDS dual-antenna mobile receiver is manufactured by Beijing Beidou Star Navigation Technology Co., Ltd. in Beijing, China. Based on the Real-Time Kinematic (RTK) accuracy measurement specification in the General Specification for BDS/GNSS RTK Receiver [18], when there are no obstacles within 15 degrees of the circumferential height angle above the mobile receiver, the positioning accuracy can reach 1.2 cm.

### 3.2. Experimental Site

**Figure 12:**
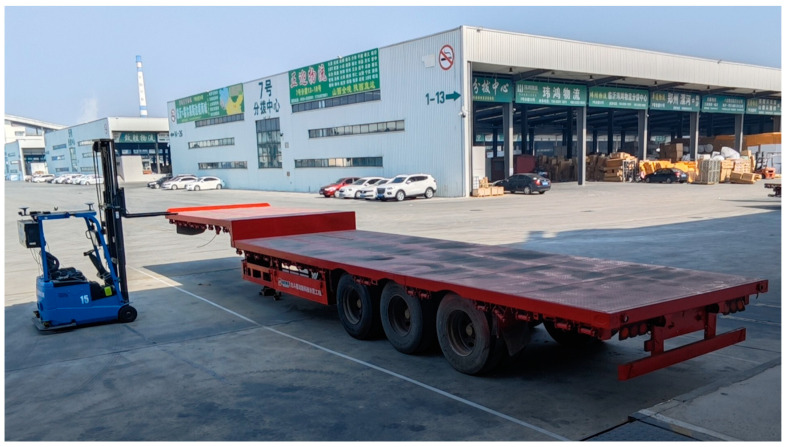
Map of the experimental field scene.

These experiments were conducted at Shunhe International Intelligent Logistics Park in Linyi City, Shandong Province. The field situation is illustrated in Figure 12. It should be noted that the method is intended for outdoor loading scenarios. During the loading process, it is essential to ensure that there are no obstructions above the forklift’s path to guarantee that the BDS receiver can acquire the satellite signal. In this case, the BDS receiver can achieve an accuracy of 1.2 cm.

On the basis of the known parking space and the length of the flatbed truck, the vertical distance from the first and second observation points to the near sideline of the parking space was set at 2 m. These points were located near the front and the rear of the truck, respectively. The unmanned forklift arrived at the first observation point to acquire and preprocess depth data, perform image segmentation, extract endpoints, collect BDS data, and calculate positions. Then, using the forklift motion control module, the unmanned forklift was directed to move to the second observation point for the aforementioned procedure. Subsequently, the rotation angle estimation was conducted after determining the position of the rear of the flatbed.

### 3.3. Flatbed Image Segmentation Datasets

**Figure 13:**
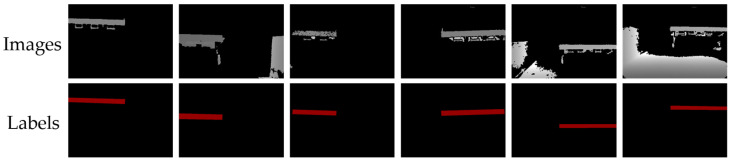
Some images with labels in the datasets.

A total of 1378 flatbed segmentation data were produced. Twenty percent of the data was allocated to the validation dataset, while the remaining eighty percent was assigned to the training dataset. The overall accuracy test of the method was conducted after training; therefore, a separate test dataset was not established. Parts of the datasets are shown in Figure 13.

During the training of the DDRNet-23-slim, the initial learning rate was set to 0.0003, the batch size was 32, and the number of training epochs was 200. The learning rate was dynamically adjusted using the cosine annealing function.

### 3.4. Parameters Setting

#### 3.4.1. Preprocessing Parameters Selection

In this scene, the segmentation threshold was set at 2.3 m, taking into account the positioning accuracy of the camera and the control precision of the unmanned forklift. This decision resulted in excluding data with a depth exceeding 2.3 m while preserving depth data equal to or less than 2.3 m. The Gaussian filter window size was set to 9 × 9.

#### 3.4.2. Line Extraction and Screening Parameters Setting

The set of straight lines with line segments longer than a specific length in the edge information was extracted. The length was set to 120 pixels for set A and 20 pixels for set B.

**Figure 14:**
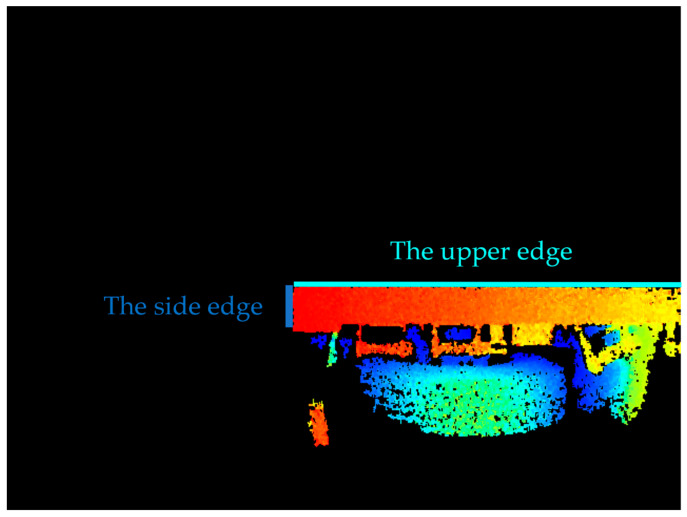
Image of the rear depth of a flatbed truck.

The upper edge contour of the flatbed truck is long and approximately transverse, while the side edge is short and approximately vertical, as illustrated in Figure 14. A set of straight lines $A_{T}$, whose angles with the upward direction (clockwise as positive and counterclockwise as negative) fell within the threshold range of [$T_{1}$, $T_{2}$], was selected from set A to align with the upper edge of the flatbed. Here, $T_{1}$ was set at 88°, and $T_{2}$ was set at 92°. When the flatbed truck is parked at an angle, the upper edge of the flatbed truck in the depth image will rotate accordingly. Therefore, the threshold range above can be appropriately increased. Similarly, a set of straight lines $B_{T}$, whose angles with the upward direction fell within the threshold value of [$T_{3}$, $T_{4}$], was selected to align with the side edges of the flatbed from the set B, where $T_{3}$ was set to be −0.5° and $T_{4}$ was set to be 0.5°.

#### 3.4.3. Number of Feature Vector Elements

The number of feature vector elements, N, was determined based on the thickness of the flatbed truck and the location of the observation point, which was set to 10 in this experiment.

## 4. Evaluation

Three measurements were taken at each endpoint using RTK, and the average value was considered as the reference data. These data were then compared with the results obtained using the method described in this paper. The absolute error (*AE*) was used to quantify the difference between an observed value and a reference value. For endpoints, *AE* represents the Euclidean distance between the observation value and the reference value, as illustrated in Formula (6). Where ${AE}_{endpoint}^{i}$ denotes the *AE* between the coordinate value of the endpoint of the ith observation and the reference value. The endpoint of the ith observation obtained with the method described in this paper was labeled with the endpoint coordinates ($r_{x}^{i}$, $r_{y}^{i}$), and the reference coordinate was labeled as ($R_{x}$, $R_{y}$). For angles, *AE* represents the absolute value of the difference between the calculated angle and the reference angle, as shown in Formula (7), where ${AE}_{angle}^{i}$ denotes the *AE* between the rotation angle of the *i*th observation and the reference value. The rotation angle of the ith observation calculated by the method in this paper was denoted as $r_{a}^{i}$, and the reference angle was denoted as ${\;R}_{a}$.

(6) $${AE}_{endpoint}^{i}=\sqrt{{\left(r_{x}^{i}-R_{x}\right)}^{2}+{\left(r_{y}^{i}-R_{y}\right)}^{2}}$$

(7) $${AE}_{angle}^{i}=\left|r_{a}^{i}-{\;R}_{a}\right|$$

Since the loading operation was carried out simultaneously on both sides of the flatbed truck, the front and rear endpoints on each side were tested 20 times. The *AE* for each observation at each endpoint and angle was calculated separately. The minimum (Formula (8)), maximum (Formula (9)), and mean (Formula (10)) of the *AE* for 20 observations at each endpoint were calculated, where n represents the number of observations.

(8) $${AE}^{min}=min\left({AE}^{1},{AE}^{2},\ldots ,{AE}^{n}\right)$$

(9) $${AE}^{max}=max\left({AE}^{1},{AE}^{2},\ldots ,{AE}^{n}\right)$$

(10) $${AE}^{mean}=\frac{1}{n}\sum_{i=1}^{n}\left({AE}^{1}+{AE}^{2}+\ldots +{AE}^{n}\right)$$

In addition, to measure the degree of dispersion of the observed data, the standard deviation (STD) was used as an evaluation indicator, as shown in Formulas (11) and (12), where ${STD}_{endpoint}$ and ${STD}_{angle}$ denote the standard deviation of the endpoint observation data and the angle calculation data, respectively. The endpoint coordinate of the ith observation obtained with the method described in this paper was labeled as ($r_{x}^{i}$, $r_{y}^{i}$). The n represents the total number of observations, and the average of n observations is denoted as ($\bar{r_{x}}$, $\bar{r_{y}}$). The rotation angle of the ith observation calculated by the method in this paper was denoted as $r_{a}^{i}$, and the mean value of the rotation angle for n observations was denoted as $\bar{r_{a}}$.

(11) $${STD}_{endpoint}=\sqrt{\frac{1}{n}\sum_{i=1}^{n}\left[{\left(r_{x}^{i}-\bar{r_{x}}\right)}^{2}+{\left(r_{y}^{i}-\bar{r_{y}}\right)}^{2}\right]}$$

(12) $${STD}_{angle}=\sqrt{\frac{1}{n}\sum_{i=1}^{n}{\left(r_{a}^{i}-\bar{r_{a}}\right)}^{2}}$$

The recognition results for each endpoint are shown in Table 3.

Evaluation indices were calculated for each endpoint and for the inclination angles on both sides. The results are shown in Table 4 and Table 5.

From the above results, the *AE* of the endpoint is less than 3 cm, and the *STD* is less than 1 cm, indicating lower dispersion of the prediction results. The angular errors *AE* and *STD* are small. The precision requirements are fully met through the automatic loading test conducted by the unmanned forklift at the logistics site.

## 5. Conclusions

This study aims to address the issue of localizing flatbed trucks and estimating rotation angles in the automatic loading process in logistics. By collecting a large amount of sample data on flatbed trucks, we have developed a high-precision positioning and rotation angle estimation algorithm for flatbed trucks using BDS and vision technology. We validated the effectiveness and reliability of this method in the automatic loading process. The results of this research are of great significance for enhancing loading efficiency, minimizing human operational errors, and advancing the field of intelligent logistics. In the future, we will continue to enhance the algorithm to better address the challenges in various loading scenarios and expand the application areas of this research.

## 6. Patents

This paper has resulted in a patent with application number CN202311047777.9, titled “Flatbed Truck Positioning Method, Installation, and Storage Medium Based on Depth Images”.

## Figures and Tables

**Table 1:** The architecture of the DDRNet-23-slim.

Stage	Output	DDRNet-23-Slim	
conv1	112 × 112	3 × 3, 32, stride 2	
conv2	56 × 56	3 × 3, 32, stride 2	
		$\left[\begin{array}{c} 3\times 3,32\\ 3\times 3,32 \end{array}\right]\times 2$	
conv3	28 × 28	$\left[\begin{array}{c} 3\times 3,64\\ 3\times 3,64 \end{array}\right]\times 2$	
conv4	14 × 14, 28 × 28	$\left[\begin{array}{c} 3\times 3,128\\ 3\times 3,128 \end{array}\right]\times 2$	$\left[\begin{array}{c} 3\times 3,64\\ 3\times 3,64 \end{array}\right]\times 2$
		Bilateral fusion	
conv5_1	7 × 7, 28 × 28	$\left[\begin{array}{c} 3\times 3,256\\ 3\times 3,256 \end{array}\right]\times 2$	$\left[\begin{array}{c} 3\times 3,64\\ 3\times 3,64 \end{array}\right]\times 2$
		Bilateral fusion	
		$\left[\begin{array}{c} 1\times 1,\;256\\ 3\times 3,256\\ 1\times 1,\;512 \end{array}\right]\times 1$	$\left[\begin{array}{c} 1\times 1,\;64\\ 3\times 3,64\\ 1\times 1,\;128 \end{array}\right]\times 1$
conv5_2	7 × 7	High-to-low fusion	
		1 × 1, 1024	
	1 × 1	7 × 7 global average pool	
		1000-d fc, softmax	

**Table 2:** Features of endpoint neighborhood pixels for various observation directions.

Case	Depth Images	Abstract Images	Endpoint Neighborhood Pixels
1	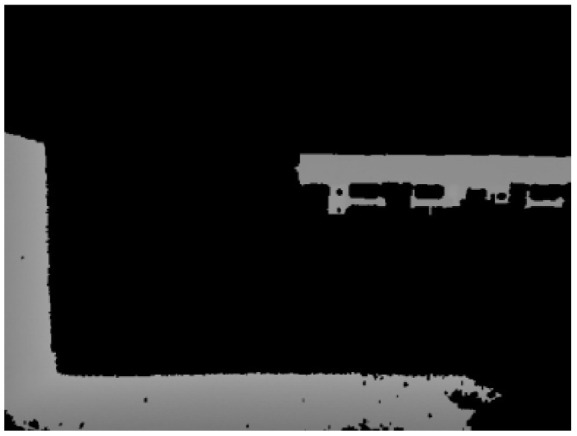	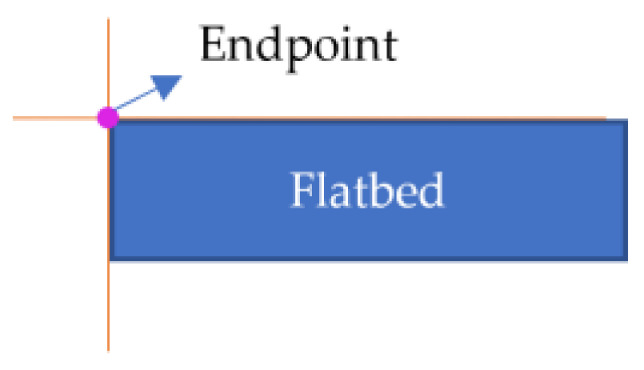	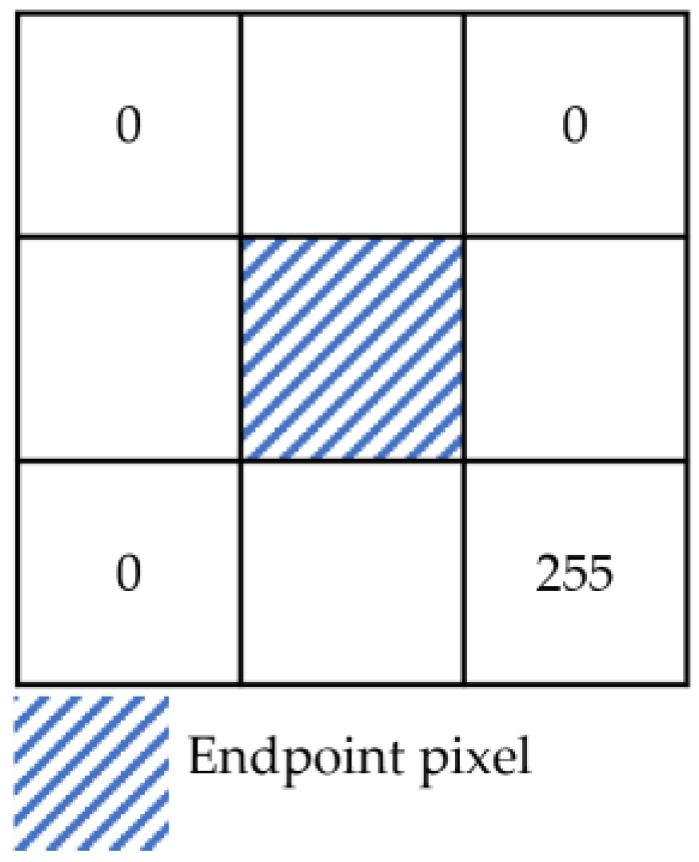
2	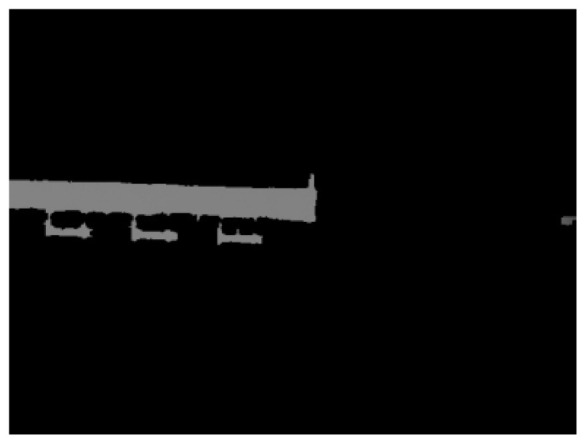	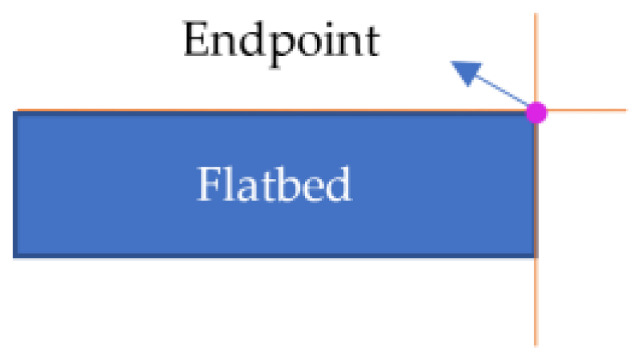	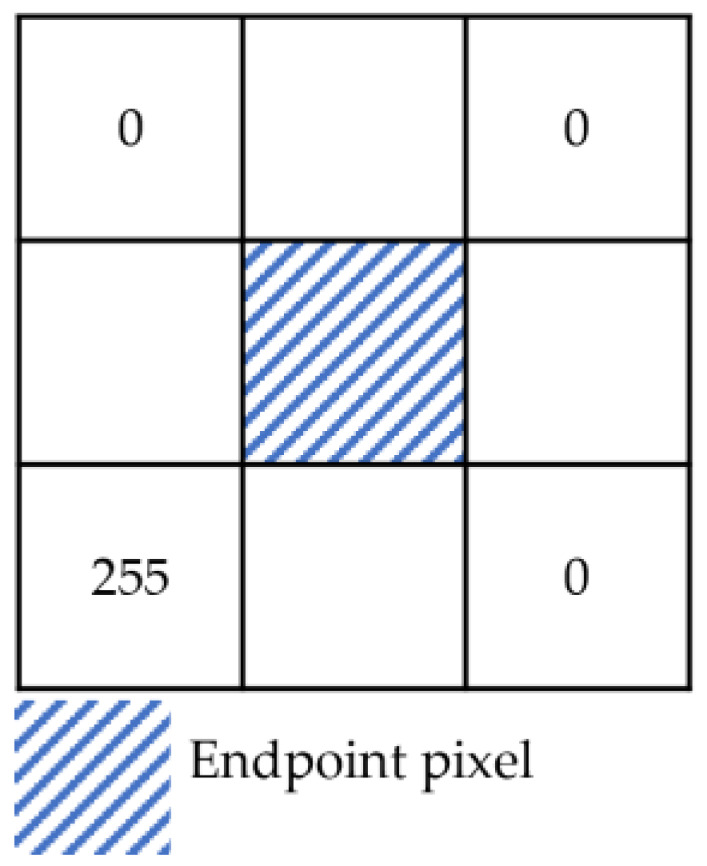

**Table 3:** Table displaying recognition results of endpoints.

Serial Number	Depth Images	Image Segmentation Results	Endpoint ^1^ Recognition Results
1	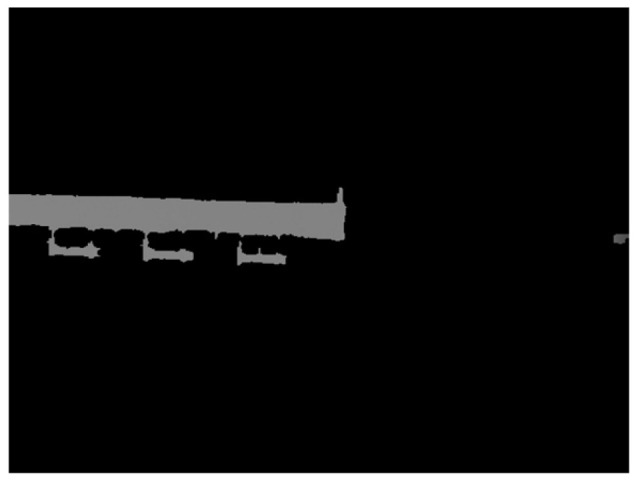	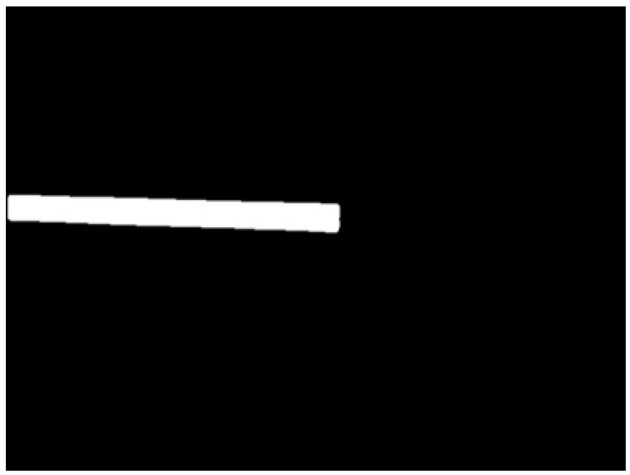	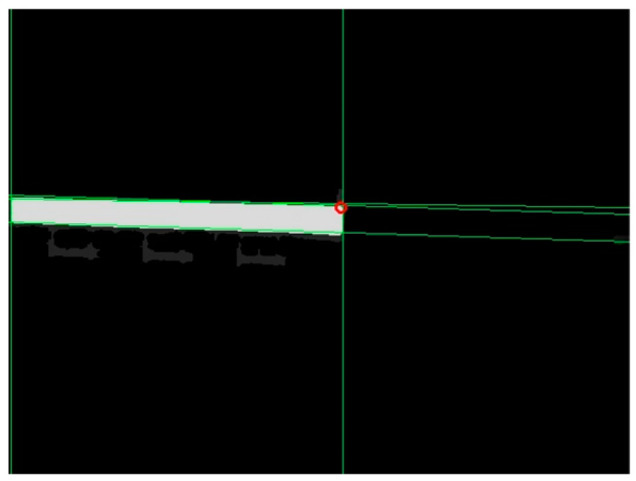
2	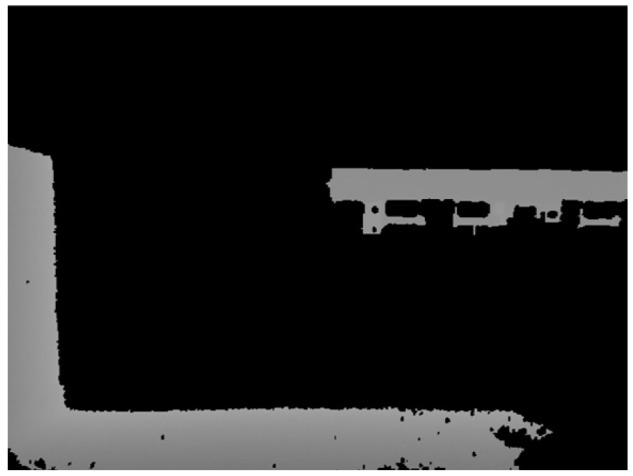	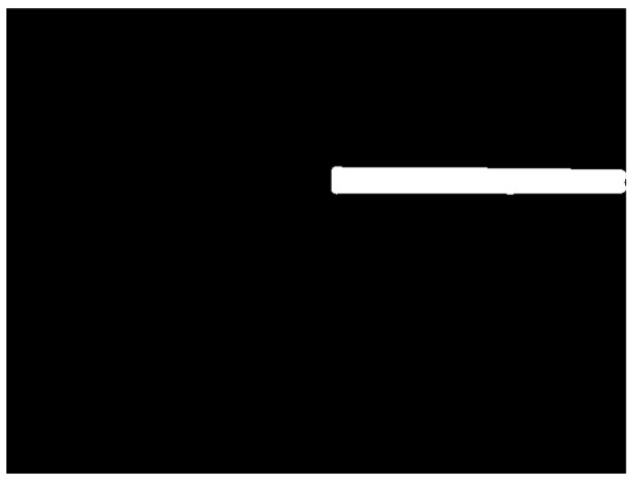	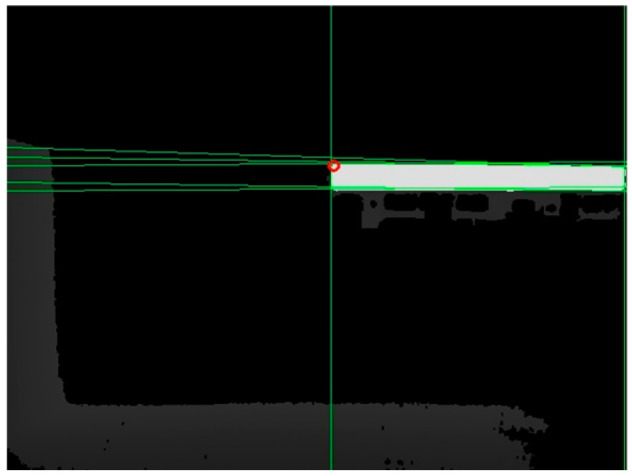
3	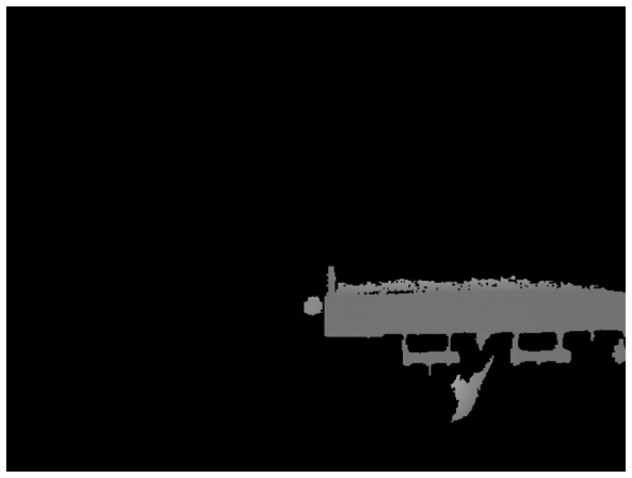	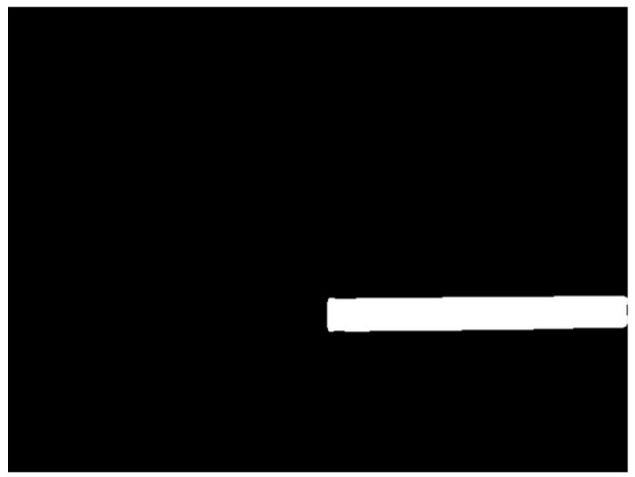	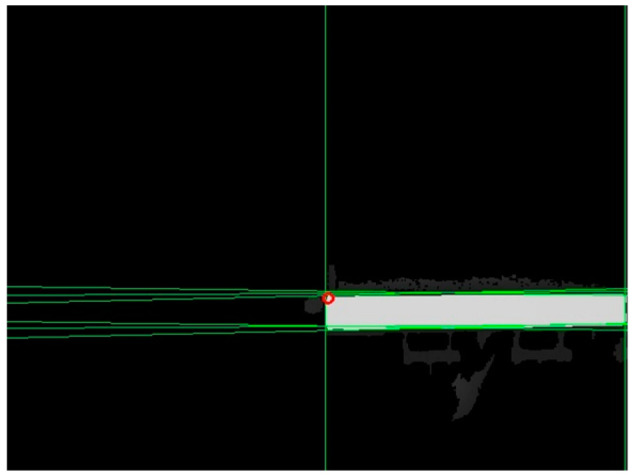
4	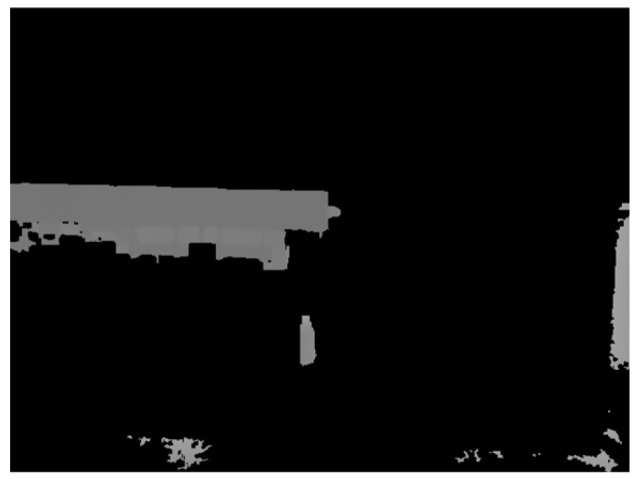	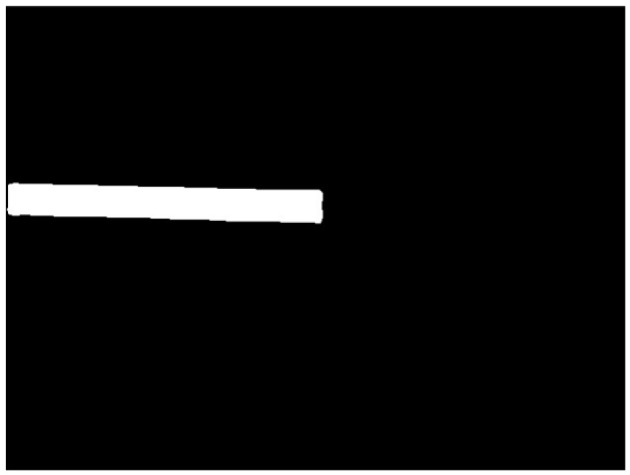	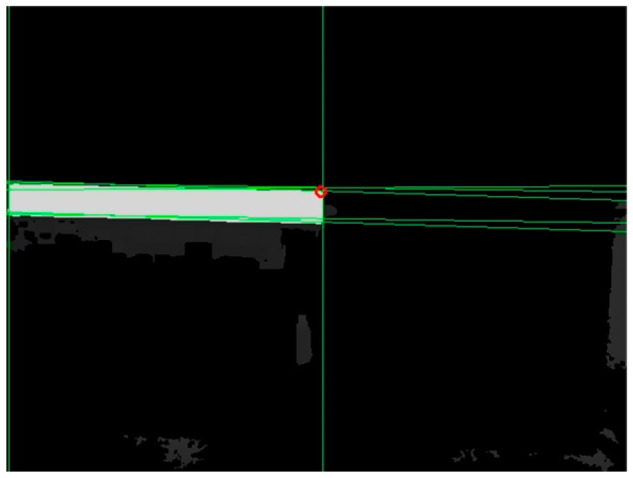

^1^ Endpoints are depicted as red circles in the images.

**Table 4:** Error between the reference and predicted coordinates of endpoints.

Endpoints	Error (mm)			
	*AE*			*STD*
	*Min*	*Max*	*Mean*	
1	24.588	28.514	26.614	6.993
2	21.230	29.285	25.790	3.978
3	21.961	27.876	25.629	2.832
4	10.871	26.450	21.775	8.934

**Table 5:** Errors between the reference and predicted rotation angles.

Angles	Error (°)			
	*AE*			*STD*
	*Min*	*Max*	*Mean*	
1	0.002	0.271	0.051	0.070
2	0.104	0.271	0.201	0.101

## Data Availability

Embargo on data due to commercial restrictions.
